# Sestrin2 inhibits YAP activation and negatively regulates corneal epithelial cell proliferation

**DOI:** 10.1038/s12276-020-0446-5

**Published:** 2020-06-12

**Authors:** Ji-Su Lee, Hwan-Woo Park, Kyong Jin Cho, Jungmook Lyu

**Affiliations:** 1 Myung-Gok Eye Research Institute, Konyang University, 158 Gwanjeodong-ro, Seo-gu, Daejeon, 35365 South Korea; 2 Department of Medical Science, Konyang University, 158 Gwanjeodong-ro, Seo-gu, Daejeon, 35365 South Korea; 3 Department of Cell Biology, Konyang University, 158 Gwanjeodong-ro, Seo-gu, Daejeon, 35365 South Korea; 4 Department of Ophthalmology, Dankook University Hospital, Dankook University College of Medicine, Dandae-ro, Dongnam-gu, Cheonan-si, Chungnam 31116 South Korea

**Keywords:** HIPPO signalling, Drug development

## Abstract

Corneal wound healing is essential for the maintenance of corneal integrity and transparency and involves a series of physiological processes that depend on the proliferation of epithelial cells. However, the molecular mechanisms that control corneal epithelial cell proliferation are poorly understood. Here, we show that Sestrin2, a stress-inducible protein, is downregulated in the corneal epithelium during wound healing and that the proliferation of epithelial basal cells is enhanced in Sestrin2-deficient mice. We also show that YAP, a major downstream effector of the Hippo signaling pathway, regulates cell proliferation during corneal epithelial wound repair and that Sestrin2 suppresses its activity. Moreover, increased levels of reactive oxygen species in the Sestrin2-deficient corneal epithelium promote the nuclear localization and dephosphorylation of YAP, activating it to enhance the proliferation of corneal epithelial cells. These results reveal that Sestrin2 is a negative regulator of YAP, which regulates the proliferative capacity of basal epithelial cells, and may serve as a potential therapeutic target for corneal epithelial damage.

## Eye injury: Healing corneal wounds

Targeting a protein that suppresses cell proliferation may speed up the healing of wounds to the eye. The cornea forms a transparent coating over the iris and pupil, protecting the eye from injury. The molecular mechanisms that modulate the wound healing response in the outer layers of the cornea are unclear. Using human cells and mouse models, Jungmook Lyu at Konyang University, Daejeon, South Korea, and co-workers have uncovered the role of two proteins, Sestrin2 and YAP, in corneal repair. YAP regulates the cell proliferation needed for repair, but high levels of Sestrin2 can suppress YAP activity. The expression of Sestrin2 is naturally reduced during wound healing, and this activates both YAP and its associated pathways, speeding up the repair process. The researchers suggest Sestrin2 could be a useful therapeutic target.

## Introduction

The corneal epithelium is the outermost layer of the cornea. It protects the eye from environmental injury and is required to maintain corneal transparency. The corneal epithelium is composed of several layers of superficial squamous cells, multiple layers of wing cells, and a single layer of basal cells, all of which are regenerated throughout life by corneal epithelial stem cells (CESCs)^1^. The repair process of corneal epithelial wound healing, caused by various injuries, involves three cellular events: basal cell proliferation, cell migration from the surrounding epithelium to the wound site, and cell differentiation into stratified layers^1–3^. These cellular events depend on complex interactions among extrinsic and intrinsic signaling pathways.

Sestrins (Sesns) are a family of highly conserved, stress-inducible proteins. Sesn1, Sesn2, and Sesn3 are found in mammals. Sesn2 has been most extensively studied as a stress response gene^4^. It is known to suppress reactive oxygen species (ROS) production through its antioxidant activity and provides cytoprotection from a variety of noxious stimuli, such as that elicited by oxidative stress, genotoxic stress, endoplasmic reticulum (ER) stress, and hypoxia^5^. Recent studies have shown that Sesn2 negatively regulates cell proliferation by inhibiting mammalian target of rapamycin complex 1 (mTORC1) signaling via the activation of AMP-activated protein kinase (AMPK)^6,7^. mTOR, a serine/threonine kinase, regulates key cellular functions, including growth, proliferation, and metabolism^8,9^. mTOR kinase exists as two large complexes, mTORC1 and mTORC2. mTORC1 phosphorylates and activates S6K, which in turn phosphorylates ribosomal protein S6^10,11^. In the cornea, mTOR signaling is known to be involved in scarring, neovascularization, and inflammation^12–15^. However, it is unclear whether Sesn2 regulates the proliferation of corneal epithelial cells.

The Hippo signaling pathway is involved in the regulation of organ size, tissue regeneration, and stem cell self-renewal^16–18^. In mammals, the Hippo pathway is composed of a core kinase cascade consisting of mammalian Ste20-like kinase 1/2 (MST1/2) and large tumor suppressor 1/2 (LATS1/2) and transcriptional coactivators. Two key downstream transcriptional coactivators, Yes-associated protein (YAP) and transcriptional coactivator with PDZ-binding motif (TAZ), induce the expression of genes that are involved in cell proliferation, survival, and migration^19–21^. When the Hippo signaling pathway is activated by upstream signals, MST1/2 are phosphorylated and activate LATS1/2. Activated LATS1/2 phosphorylate the transcription coactivator YAP at Ser127, which accumulates in the cytoplasm and is thus excluded from the nucleus^22–26^. Inactivation of the Hippo signaling pathway induces the dephosphorylation of YAP and its translocation into the nucleus to promote TEAD binding and the activation of gene transcription^25^. Recent studies have revealed that YAP is expressed in corneal basal epithelial cells and plays an essential role in maintaining their proliferation^27^. However, the underlying mechanism of YAP regulation in corneal wound healing remains unknown.

In this study, we show that Sesn2 deficiency promotes the proliferation of corneal epithelial cells during corneal wound healing in vitro and in vivo. We also show that YAP is activated in Sesn2-deficient corneal epithelial cells, which results in an increase in ROS levels. These results demonstrate that Sesn2 suppresses YAP activity and negatively regulates corneal epithelial cell proliferation.

## Materials and methods

### Cell culture and lentiviral infection

Human corneal epithelial (hCET) cells were provided by Kaoru Araki-Sasaki (Osaka University, Osaka, Japan). The cells were cultured at 37 °C in Dulbecco’s modified Eagle’s medium (DMEM)/F-12 (1:1; Corning, NY, USA) supplemented with 5% fetal bovine serum (Corning), 1× penicillin/streptomycin (WELGENE, Gyeongsan, Korea), 500 ng/ml hydrocortisone (Sigma-Aldrich, St Louis, MO, USA) and 30 ng/ml cholera toxin (Sigma-Aldrich). For Sesn2- and/or YAP-knockdown hCET cells, lentiviruses expressing *Sesn2* shRNA, *YAP* shRNA, or wild-type *Sesn2* were generated as previously described^5^ and transfected into the hCET cells. The *Sesn2* shRNA and wild-type *Sesn2* lentiviral plasmids were kindly provided by Andrei V. Budanov (Trinity College, Dublin, Ireland), and the *YAP* shRNA lentiviral plasmid (#42540) was obtained from Addgene (Cambridge, MA, USA).

### In vivo and in vitro wound healing assays

*Sesn2*^−/−^ mice with a CL57BL/6 background were kindly provided by Dr. Seo Goo Rhee (Yonsei University, South Korea). Eight-week-old *Sesn2*^−/−^ and *Sesn2*^+/+^ mice were administered general anesthesia with Zoletil (Virbac, Carros, France) and Rompun (Bayer, Seoul, South Korea). A circular defect was produced in the central corneal epithelium using a sterile disposable biopsy punch with a diameter of 2 or 2.5 mm (Kai, Tokyo, Japan). The corneal epithelial defects were stained with fluorescein and photographed at 0 and 24 h for the 2 mm defect and at 0, 24, 48, 72, and 96 h for the 2.5 mm defect.

For the in vitro wound healing assay, Ibidi culture inserts (Ibidi GmbH, Münich, Germany) were placed at the bottom of the wells in a 6-well plate. hCET cells were seeded into each well and incubated for 24 h. Thereafter, the Ibidi culture inserts were removed to create a wound. The cells were photographed at 0, 12, and 24 h. Wound healing was measured by determining the percentage of the wound area.

### Immunostaining and western blotting

For immunostaining, cells grown on coverslips were fixed in 4% paraformaldehyde and permeabilized with 0.1% Triton X-100. The fixed cells and corneal sections were incubated with a blocking solution containing 1% bovine serum albumin (BSA) and 5% normal goat serum for 1 h and incubated overnight at 4 °C with anti-pS6 and anti-Yap antibodies. Thereafter, the cells were rinsed with PBS, incubated with secondary antibodies (Alexa Fluor 488) at room temperature for 1 h, and counterstained with Hoechst 33342. Images were acquired using a fluorescence microscope with an AxioCam camera (Zeiss, Jena, Germany).

For western blotting, proteins were extracted in lysis buffer (25 mM Tris-HCl (pH 7.4), 150 mM sodium chloride, 5 mM EDTA, 1× Triton X-100, 10% glycerol, 10 mM sodium pyrophosphate, 10 mM β-glycerophosphate, 1 mM sodium orthovanadate, 1× protease inhibitor, 10 mM sodium fluoride, and 1 mM phenylmethylsulfonyl fluoride). The concentration of proteins in the total cell extracts was determined using a bicinchoninic acid assay (Thermo Fisher Scientific, Waltham, MA, USA). Thereafter, equal amounts of proteins were separated by 10% SDS-PAGE and transferred to polyvinylidene difluoride membranes, which were then blocked with 5% nonfat dry milk or 5% BSA in TBS with 0.02% Tween-20 for 1 h. The membranes were incubated overnight with primary antibodies at their optimal concentration at 4 °C. The next day, the membranes were washed with TBST three times for 5 min each time and incubated with horseradish peroxidase-conjugated secondary antibody for 1.5 h at room temperature. The immunoreactive bands were visualized using an enhanced chemiluminescence detection kit (Bio-Rad, Hercules, CA, USA). The following antibodies were used: phospho-YAP (Cell Signaling, Cat. 4911), YAP (Cell Signaling, Cat. 14074), Sestrin2 (provided by Jun Hee Lee, University of Michigan), phospho-S6 (Cell Signaling, Cat. 2211), and S6 (Santa Cruz, Cat. 74459).

### BrdU and EdU incorporation assays

For in vivo proliferation analysis, *Sesn2*^+/+^ and *Sesn2*^−/−^ mice were injured and injected intraperitoneally with 5-bromo-2′-deoxyuridine (BrdU) (100 mg/kg). The mouse eyes were collected 48 h after injection and fixed. Corneal sections were incubated in 1 M HCl for 30 min at 37 °C and washed in 0.5 mM borate buffer before incubation with an anti-BrdU antibody. Cell proliferation was assessed by immunostaining BrdU (Abcam, Cambridge, UK). Nuclei were stained with Hoechst 33342.

For in vitro proliferation analysis, hCET cells were grown on coverslips in culture medium, and 1 µM 5-ethyl-2′-deoxyuridine (EdU) was added. After incubation with EdU for 6 h, +cells were washed three times with PBS, fixed in 4% PFA, and incubated in 200 µl of Click-iT reaction mix (Click-iT EdU Alexa Fluor 488 Imaging Kit; Invitrogen, Carlsbad, CA, USA) for 30 min. Cell proliferation was measured by determining the amount of EdU incorporation in each cell.

### Luciferase assay

For the luciferase assay, hCET cells expressing *Sesn2* shRNA or control shRNA were seeded in 24-well plates. Cells were transfected with the YAP reporter 8xGTIIC-lux (Addgene, Cambridge, USA) and an internal control, pRL-TK. The cells were harvested 24 h after transfection and analyzed using a dual-luciferase reporter assay kit (Promega, Wisconsin, USA).

### ROS detection

Oxidation-sensitive fluorescent dye dihydroethidium (DHE) was used to assess intracellular ROS levels. Injured corneal sections from *Sesn2*^+/+^ and *Sesn2*^−/−^ mice were washed in PBS and incubated in 10 μM DHE for 30 min. Then, the corneal sections were washed in PBS and mounted onto glass slides before images were acquired.

### Cell cycle assay

Cell cycle analyses were performed using fluorescence-activated cell sorting (FACS). hCET cells expressing control shRNA or *Sesn2* shRNA were harvested from a 6-well plate and fixed overnight in 70% ethanol at 20 °C. After centrifugation at 800 rcf for 3 min, the pellet was resuspended in PBS and stained with a cell cycle solution (Tali® Cell Cycle kit; Invitrogen, Carlsbad, CA, USA) for 30 min under dark conditions. The cell cycle profile was analyzed using a flow cytometer (NovoCyte, ACEA Biosciences, San Diego, CA, USA).

### Quantitation of nuclear YAP

To determine whether YAP translocated into the nucleus of the corneal epithelial cells in the *Sesn2*^−/−^ mice treated with NAC or DMSO, mouse corneal sections were immunostained with an anti-YAP antibody. The values obtained from at least two experiments were averaged and are presented as the means ± standard deviation (SD). Hoechst 33258 was used for nuclear staining. Fluorescence images were acquired using a fluorescence microscope with an AxioCam camera (Zeiss, Jena, Germany). For each nucleus, the fluorescence intensity of the anti-YAP secondary antibody was measured using ZEN software (Zeiss). A minimum of 45 corneal epithelial cells were examined.

## Results

### Sesn2 negatively regulates corneal epithelial cell proliferation

**Fig. 1:**
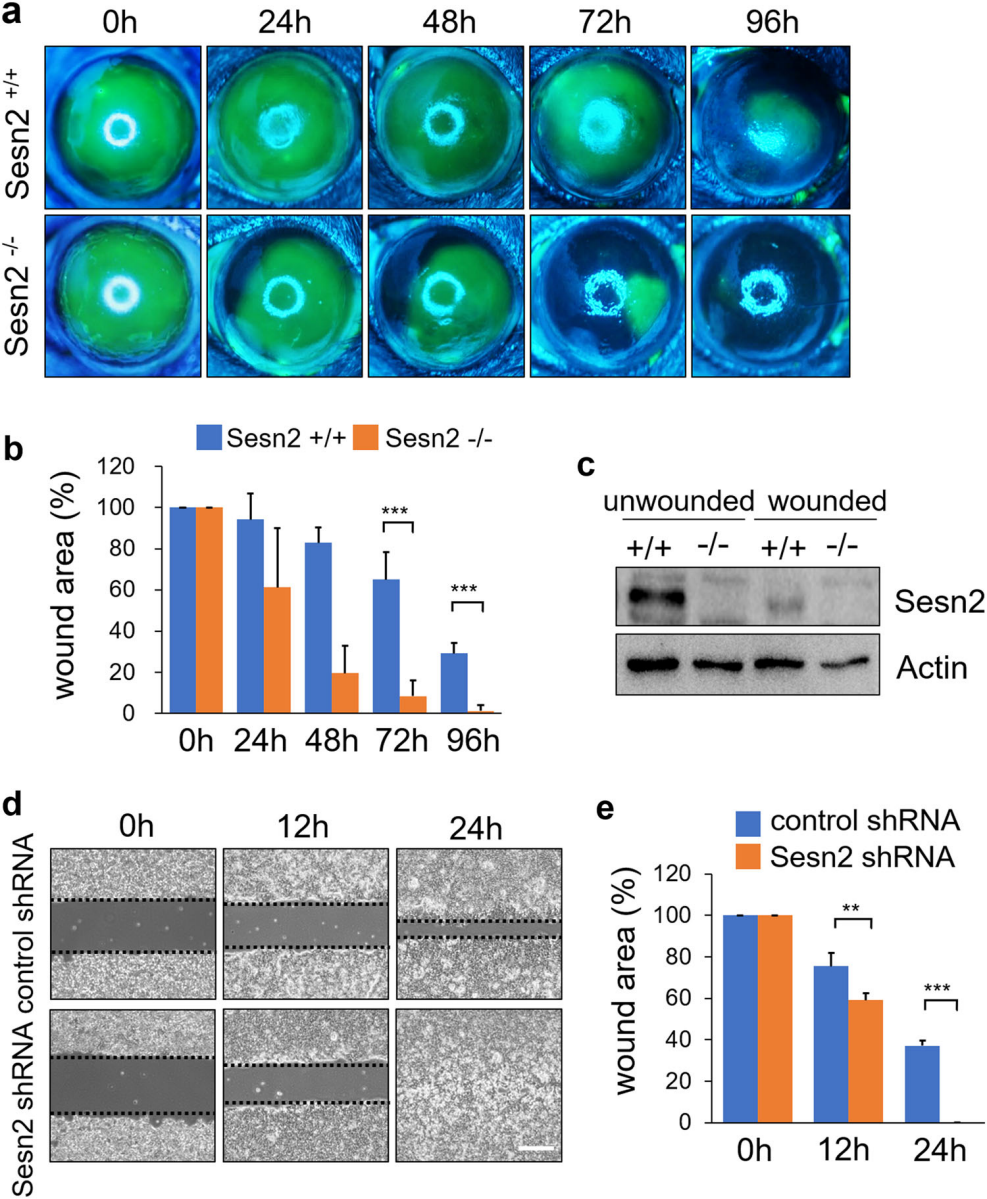
Sesn2 deficiency enhances corneal wound healing. **a** Representative photographs of the fluorescein-stained corneas of *Sesn2*^−/−^ and *Sesn2*^+/+^ mice at 0, 24, 48, 72, and 96 h after injury using a 2.5 mm punch. The fluorescein-stained area represents the corneal epithelial wound area. By 96 h, the corneal wound area in *Sesn2*^−/−^ mice was healed, whereas that of *Sesn2*^+/+^ mice remained open. **b** Quantitative analysis of the corneal wound area of *Sesn2*^−/−^ and +/+ mice. A total of 12 eyes from *Sesn2*^−/−^ and *Sesn2*^+/+^ mice were used to measure the epithelial wound area size. The wound area was significantly smaller in *Sesn2*^−/−^ mice than in *Sesn2*^+/+^ mice. **c** Corneal tissues from *Sesn2*^−/−^ and *Sesn2*^+/+^ mice were lysed 48 h after injury and subjected to western blotting with Sesn2 and actin antibodies. **d** In vitro wound healing assays of hCET cells expressing *Sesn2* shRNA and control shRNA. hCET cells expressing *Sesn2* shRNA or control shRNA were seeded on both sides of a wound chamber and allowed to attach for 12 h. The chamber was removed, and the wound areas were photographed immediately at 0, 12, and 24 h. Dotted lines indicate wound borders at the beginning of the assay. **e** Quantitative analysis of the wound areas of hCET cells expressing *Sesn2* shRNA and control shRNA at 0, 12, and 24 h. The rate of wound closure in hCET cells expressing *Sesn2* shRNA was significantly higher than in hCET cells expressing control shRNA. Error bars represent the means ± SD of three independent experiments. Two-tailed Student’s *t*-test (**p < 0.01, ***p < 0.001). Scale bar, 300 µm.

To investigate the function of Sesn2 in corneal wound healing, corneas of *Sesn2*^+/+^ and *Sesn2*^−/−^ mice were first denuded of their epithelium, and corneal wound healing was monitored by fluorescein staining for 4 days. The corneal wound area in *Sesn2*^−/−^ mice was almost completely healed within 96 h, whereas that of *Sesn2*^+/+^ mice remained unhealed (Fig. 1a). The percentage of wound area was determined in *Sesn2*^−/−^ and *Sesn2*^+/+^ mice. The results revealed that *Sesn2*^−/−^ mice had a significantly faster rate of wound closure than *Sesn2*^+/+^ mice (Fig. 1b). Western blot analysis of corneal epithelial lysates revealed that Sesn2 protein expression was decreased in the injured cornea compared to that in the normal cornea of *Sesn2*^+/+^ mice (Fig. 1c). These results suggest that Sesn2 can inhibit corneal epithelial wound healing. To address this possibility, hCET cells transduced with lentiviruses expressing *Sesn2* shRNA or control shRNA were seeded into wound assay chambers and monitored for 24 h after wounding. At 12 and 24 h, the wound closure rate of hCET cells expressing *Sesn2* shRNA was significantly higher than that of those expressing control shRNA (Fig. 1d, e). In addition, when wild-type *Sesn2* was re-expressed in Sesn2-deficient hCET cells, wound closure was delayed (Supplementary Fig. S1). Taken together, these results suggest that Sesn2 deficiency enhances corneal epithelial wound healing.

**Fig. 2:**
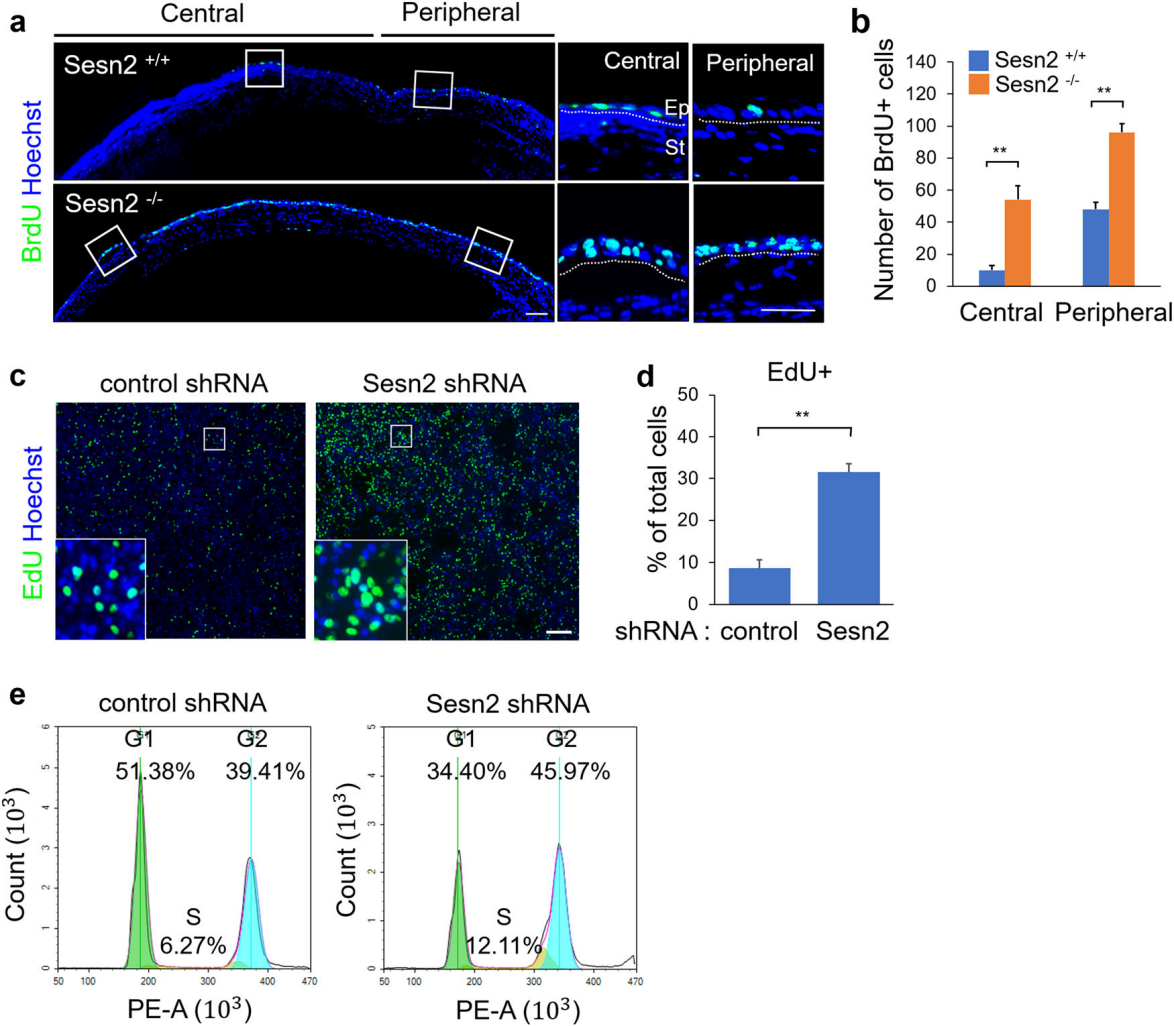
Sesn2 deficiency promotes corneal epithelial cell proliferation. **a** BrdU was injected into *Sesn2*^−/−^ and *Sesn2*^+/+^ mice after injury, and the mice were sacrificed 48 h. Corneal sections were stained using an anti-BrdU antibody (green). Hoechst dye was used as a counterstain (blue). BrdU-positive cells were observed in the central and peripheral regions of corneal epithelial layers and were primarily detected in *Sesn2*^−/−^ mice. Dotted lines indicate the basement membrane. **b** Quantitative analysis of BrdU-positive cells in the corneal epithelium of *Sesn2*^−/−^ and *Sesn2*^+/+^ mice after injury. **c** EdU incorporation assay of hCET cells expressing *Sesn2* shRNA or control shRNA. Cells were incubated with 10 µM EdU for 4 h. **d** Percentage of EdU-positive cells. The number of EdU-positive Sesn2-deficient hCET cells was significantly increased. **e** Distribution of cells in different cell cycle phases. The proportion of Sesn2-deficient hCET cells in the S and G2 phases of the cell cycle was higher than that of control cells. Error bars represent the means ± SD of three independent experiments. Two-tailed Student’s *t*-test (**p < 0.01). Scale bars, 50 µm in (**a**); 100 µm in (**c**).

To determine whether Sesn2 regulates the proliferative capacity of epithelial cells during corneal wound healing, we injected *Sesn2*^+/+^ and *Sesn2*^−/−^ mice with bromodeoxyuridine (BrdU) and mechanically induced corneal epithelial wounds. BrdU-incorporated cells were identified by immunostaining using an anti-BrdU antibody 48 h of injury. BrdU-positive cells were detected in both the central and peripheral regions of the corneal epithelium (Fig. 2a). Moreover, the number of BrdU-positive cells in the cornea of *Sesn2*^−/−^ mice was significantly higher than that in the cornea of *Sesn2*^+/+^ mice (Fig. 2a, b). In addition, the number of EdU-positive cells was significantly increased in cultures expressing *Sesn2* shRNA compared to cultures expressing control shRNA (Fig. 2c, d). To further confirm the effect of Sesn2 on the proliferative potential of hCET cells, the distribution of hCET cells expressing control shRNA or *Sesn2* shRNA in different phases of the cell cycle was analyzed. The proportion of *Sesn2* shRNA-expressing hCET cells in the S/G2 phase was higher than that of control shRNA-expressing hCET cells (Fig. 2e). These results suggest that Sesn2 deficiency can facilitate the proliferation of corneal epithelial cells by regulating the S/G2 phase of the cell cycle.

### Sesn2 deficiency increases mTOR signaling activity

**Fig. 3:**
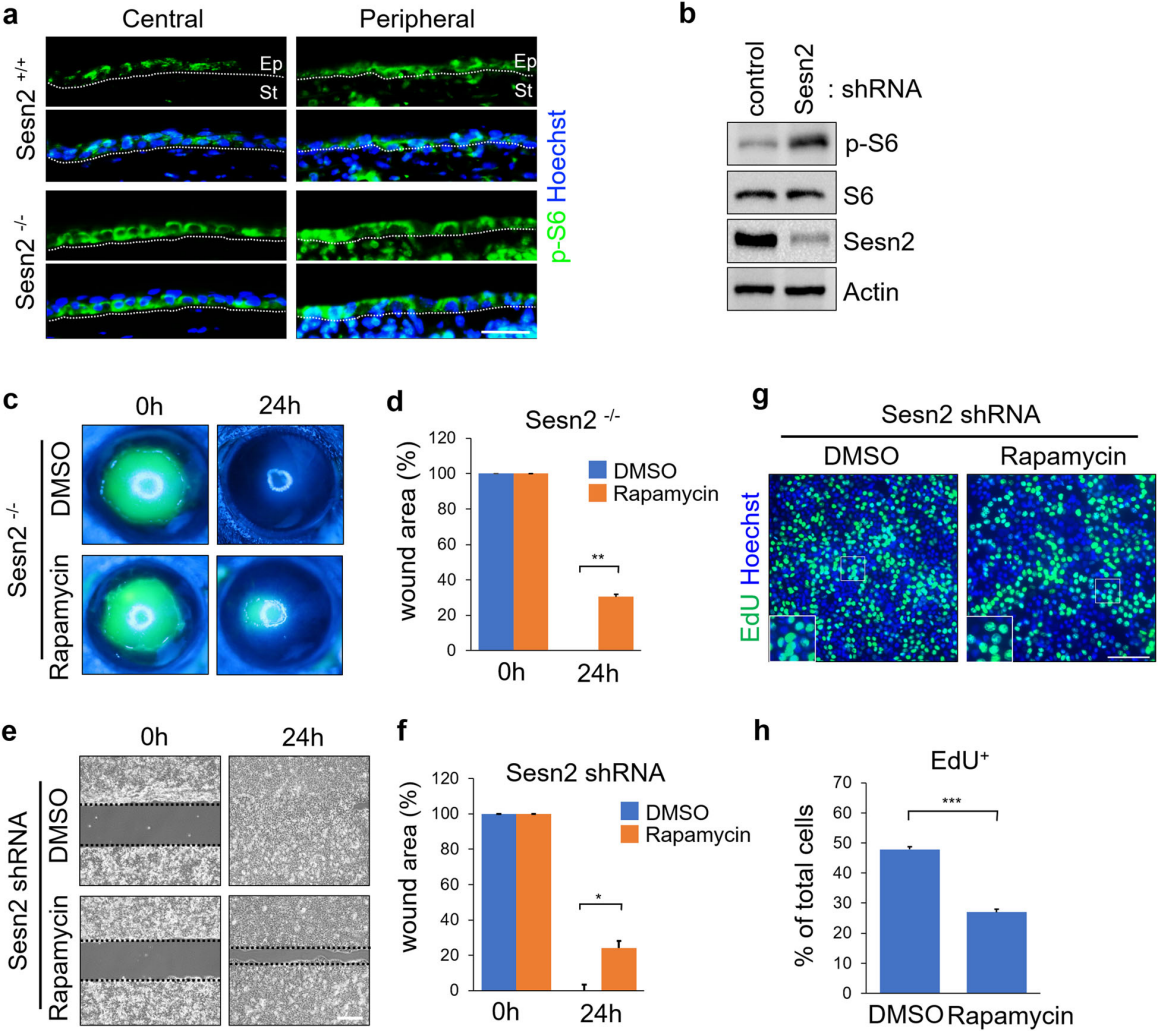
Sesn2 deficiency increases mTOR signaling activity. **a** Sections of injured corneas from *Sesn2*^−/−^ and *Sesn2*^+/+^ mice were immunostained using an anti-phospho-S6 antibody. Phospho-S6 expression was increased in the corneal epithelium of *Sesn2*^−/−^ mice compared to that in the corneal epithelium of *Sesn2*^+/+^ mice. Dotted lines indicate the basement membrane. **b** hCET cells expressing *Sesn2* shRNA or control shRNA were subjected to western blotting with phospho-S6, S6, Sesn2, and actin antibodies. **c** In vivo corneal epithelial wound healing in *Sesn2*^−/−^ mice after injury using a 2 mm punch. Each cornea was treated with 100 nM rapamycin or DMSO after injury. At 24 h, the wound area of rapamycin-treated *Sesn2*^−/−^ mice remained open, whereas that of DMSO-treated *Sesn2*^−/−^ mice had healed. **d** Quantitative analysis of the wound area of rapamycin- and DMSO-treated *Sesn2*^−/−^ mice. Wound closure in rapamycin-treated *Sesn2*^−/−^ mice was significantly delayed compared to that in DMSO-treated *Sesn2*^−/−^ mice. **e** In vitro wound healing assay of hCET cells expressing *Sesn2* shRNA treated with 100 nM rapamycin or DMSO. **f** Quantitative analysis of wound area showing that the rate of wound closure in rapamycin-treated cells was significantly delayed compared to that in DMSO-treated cells. **g** EdU incorporation assay of Sesn2-deficient hCET cells treated with rapamycin or DMSO. **h** The number of EdU-positive cells was decreased in rapamycin-treated cultures compared to DMSO-treated cultures. Error bars represent the mean ± SD of three independent experiments. Two-tailed Student’s *t*-test (*p < 0.05, **p < 0.01, ***p < 0.001). Scale bars, 50 µm in (**a**, **g**); 300 µm in (**e**).

We next sought to determine whether Sesn2 regulates mTOR signaling activity in corneal epithelial wound healing. The sections of injured corneas from *Sesn2*^+/+^ and *Sesn2*^−/−^ mice were immunostained with an antibody against the phosphorylated S6 ribosomal protein, a marker of mTORC1 activity. Phospho-S6 proteins were detected in the basal cells of the corneal epithelium (Fig. 3a). The expression of phospho-S6 protein in the central and peripheral corneal regions of *Sesn2*^−/−^ mice was higher than that in these regions of the *Sesn2*^+/+^ mice. Moreover, western blotting revealed that pS6 expression was increased in the hCET cells expressing *Sesn2* shRNA compared to that of the cells expressing control shRNA (Fig. 3b). To evaluate whether mTOR signaling promotes wound healing in Sesn2-deficient corneas, the corneal epithelium of *Sesn2*^−/−^ mice was mechanically denuded and treated with rapamycin, an inhibitor of mTORC1, or DMSO. Wound closure was delayed in the rapamycin-treated *Sesn2*^−/−^ mice, whereas the wound area was completely healed in DMSO-treated *Sesn2*^−/−^ mice 24 h after injury (Fig. 3c). The wound area percentage was quantitated; the rate of corneal epithelial wound closure in rapamycin-treated *Sesn2*^−/−^ mice was significantly delayed compared to that in DMSO-treated counterparts (Fig. 3d). We also observed delayed wound closure in rapamycin-treated *Sesn2*^+/+^ mice (Supplementary Fig. S2a, b), although the percentage of reduction in the wound closure rate was markedly higher in *Sesn2*^−/−^ mice than in *Sesn2*^+/+^ mice. In addition, the in vitro wound assay using Sesn2-deficient hCET cells treated with rapamycin or DMSO produced results similar to those found for the mice (Fig. 3e, f). However, no significant difference in the percentage of wound area was observed between the control shRNA-expressing hCET cells treated with rapamycin and DMSO (Supplementary Fig. S2c, d). This finding indicates that rapamycin affects corneal epithelial wound healing in a Sesn2-dependent manner. To test the effect of rapamycin on the proliferation of corneal epithelial cells, we treated hCET cells expressing *Sesn2* shRNA with rapamycin and DMSO and performed an EdU incorporation assay. Rapamycin treatment significantly decreased the number of EdU-positive cells (Fig. 3g, h). Taken together, these results demonstrate that Sesn2 deficiency activates mTOR signaling and promotes the proliferation of corneal epithelial cells. Therefore, mTOR signaling promotes corneal wound healing and is negatively regulated by Sesn2.

### Sesn2 negatively regulates YAP activity

**Fig. 4:**
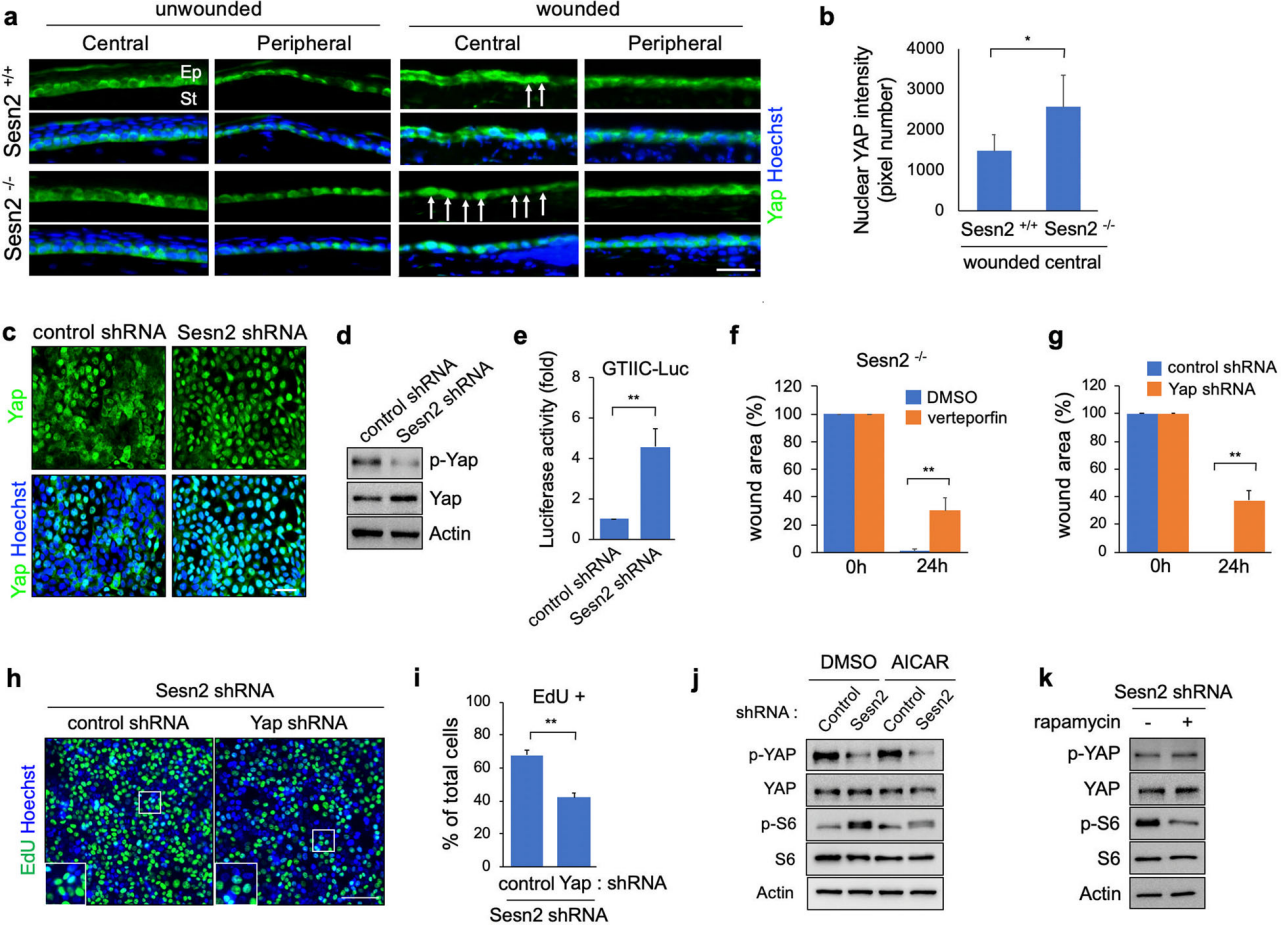
Sesn2 negatively regulates YAP activity. **a** Sections of injured and normal corneas from *Sesn2*^−/−^ and *Sesn2*^+/+^ mice were immunostained using an anti-YAP antibody. YAP is expressed in the basal layer of the peripheral and central corneal epithelium. White arrows indicate the cells showing YAP nuclear localization. **b** Fluorescence intensity of nuclear YAP in the epithelial cells of the central cornea from *Sesn2*^−/−^ and *Sesn2*^+/+^ mice. **c** Sesn2 deficiency increased YAP nuclear localization. hCET cells expressing *Sesn2* shRNA or control shRNA were immunostained using an anti-YAP antibody. Hoechst dye was used as a counterstain. **d** Sesn2 deficiency increased YAP activity. hCET cells expressing *Sesn2* shRNA or control shRNA were subjected to western blotting with antibodies against phospho-YAP, YAP, and actin. **e** hCET cells expressing *Sesn2* shRNA or control shRNA were transfected with the GTIIC-Lux reporter. Luciferase activity in each sample was measured as described in the Materials and Methods section. **f** Quantitative analysis of corneal epithelial wound healing in *Sesn2*^−/−^ mice after injury using a 2 mm punch. Each cornea was treated with 20 µM verteporfin or DMSO after injury. The wound area was significantly smaller in DMSO-treated corneas than in verteporfin-treated corneas. **g** Quantitative analysis of the in vitro wound healing of Sesn2-deficient hCET cells expressing *YAP* shRNA or control shRNA. Wound closure in hCET cells expressing *YAP* shRNA was significantly delayed compared to that in hCET cells expressing control shRNA. **h** EdU incorporation assay of Sesn2-deficient hCET cells expressing *YAP* shRNA or control shRNA. **i** Percentage of EdU-positive cells. YAP deficiency decreased the proliferation of corneal epithelial cells. **j** hCET cells expressing *Sesn2* shRNA or control shRNA were treated with 0.5 mM AICAR or DMSO and subjected to western blotting with phospho-YAP, YAP, phospho-S6, S6, and actin antibodies. **k** Western blot analysis of cell lysates from *Sesn2* shRNA-expressing hCET cells treated with rapamycin or DMSO. Rapamycin treatment did not affect phospho-Yap expression. Error bars represent the means ± SD of three or four independent experiments. Two-tailed Student’s *t*-test (*p < 0.05, **p < 0.01). Scale bars, 50 µm in (**c**, **h**); 100 µm in (**a**).

Sesn2 can potentially regulate the Hippo signaling pathway because AMPK and mTORC1 are known to regulate YAP activity. To investigate whether the loss of Sesn2 affects YAP activity in corneal epithelial cells, we analyzed the expression of YAP protein in normal and injured corneas of *Sesn2*^+/+^ and *Sesn2*^−/−^ mice. Immunostaining with an anti-YAP antibody revealed that YAP protein is expressed in the basal layer of the corneal epithelium (Fig. 4a). In the normal corneas, YAP protein was mainly detected in the cytoplasm of basal epithelial cells in the central region, whereas its protein was expressed in both the cytoplasm and nucleus of epithelial cells in the peripheral region. Interestingly, the injured corneas of *Sesn2*^+/+^ mice exhibited increased nuclear localization of YAP protein in the basal epithelial cells in the central region (Fig. 4a, arrows) but not in the epithelial cells in the peripheral region. The number of basal epithelial cells exhibiting nuclear localization of YAP in the central region was increased in the injured cornea of *Sesn2*^−/−^ mice. Fluorescence intensity analysis corroborated this finding by showing that nuclear YAP was detected more frequently in the corneal epithelial cells of *Sesn2*^−/−^ mice than in the epithelial cells of *Sesn2*^+/+^ mice (Fig. 4b). In addition, the number of cells showing predominant nuclear localization of YAP in cultures expressing *Sesn2* shRNA was higher than in those expressing control shRNA (Fig. 4c). YAP protein is dephosphorylated at the serine 127 residue upon translocation into the nucleus and is subsequently activated. To determine whether Sesn2 negatively regulates YAP activation, hCET cells transduced with lentiviruses expressing *Sesn2* shRNA or control shRNA were subjected to western blotting using an anti-phospho-YAP (serine 127) antibody. As expected, the expression of phosphorylated YAP protein in cultures expressing *Sesn2* shRNA was lower than that in cultures expressing control shRNA (Fig. 4d). In addition, re-expression of wild-type Sesn2 in Sesn2-deficient hCET cells resulted in an increase in the level of phosphorylated YAP protein (Supplementary Fig. S3a). Furthermore, the GTIIC reporter activity of YAP was increased in the cultured cells expressing *Sesn2* shRNA compared to those expressing control shRNA (Fig. 4e). These results demonstrate that Sesn2 deficiency increases YAP activity.

To further investigate whether YAP is required for Sesn2 deficiency-mediated corneal wound healing, the mechanically denuded corneal epithelium in *Sesn2*^−/−^ mice was treated with verteporfin, a porphyrin compound that blocks the interaction between YAP and TEAD and represses YAP function^28^, or DMSO. The rate of corneal epithelial wound closure was significantly delayed in verteporfin-treated corneas compared to DMSO-treated corneas (Fig. 4f, Supplementary Fig. S3b). We performed an in vitro wound assay using Sesn2-deficient hCET cells transduced with lentiviruses expressing *YAP* shRNA or control shRNA and observed delayed wound closure in cultures expressing *YAP* shRNA (Supplementary Fig. S3c). The wound area was completely healed in cultures expressing control shRNA after 24 h. The percentage of the wound area was quantitated, and the results showed that *YAP* deficiency decreased the rate of wound closure (Fig. 4g). Next, we tested whether YAP promotes the proliferation of corneal epithelial cells. The number of EdU-positive cells in Sesn2-deficient hCET cells expressing YAP shRNA was significantly decreased as compared with that in Sesn2-deficient hCET cells expressing control shRNA (Fig. 4h, i). These results indicate that YAP is required to regulate the proliferation of hCET cells in Sesn2 deficiency-mediated corneal wound healing.

To determine whether AMPK regulates YAP activity, we treated hCET cells expressing *Sesn2* shRNA or control shRNA with 5-aminoimidazole-4-carboxamideribonucleotide (AICAR), an AMPK activator, or DMSO. Western blot analysis showed that the expression of phospho-S6 protein was decreased in AICAR-treated cultured cells expressing *Sesn2* shRNA compared to DMSO-treated cultures expressing *Sesn2* shRNA (Fig. 4j). However, there was no difference in the expression of phospho-YAP protein between AICAR-treated and DMSO-treated hCET cells expressing *Sesn2* shRNA. Moreover, rapamycin treatment did not affect YAP activity in hCET cells expressing *Sesn2* shRNA, while the expression of phospho-S6 protein was decreased in these cells (Fig. 4k). These results suggest that Sesn2 regulates YAP activation via an AMPK- and mTORC1-independent mechanism.

### Sesn2 deficiency activates YAP by regulating ROS production

**Fig. 5:**
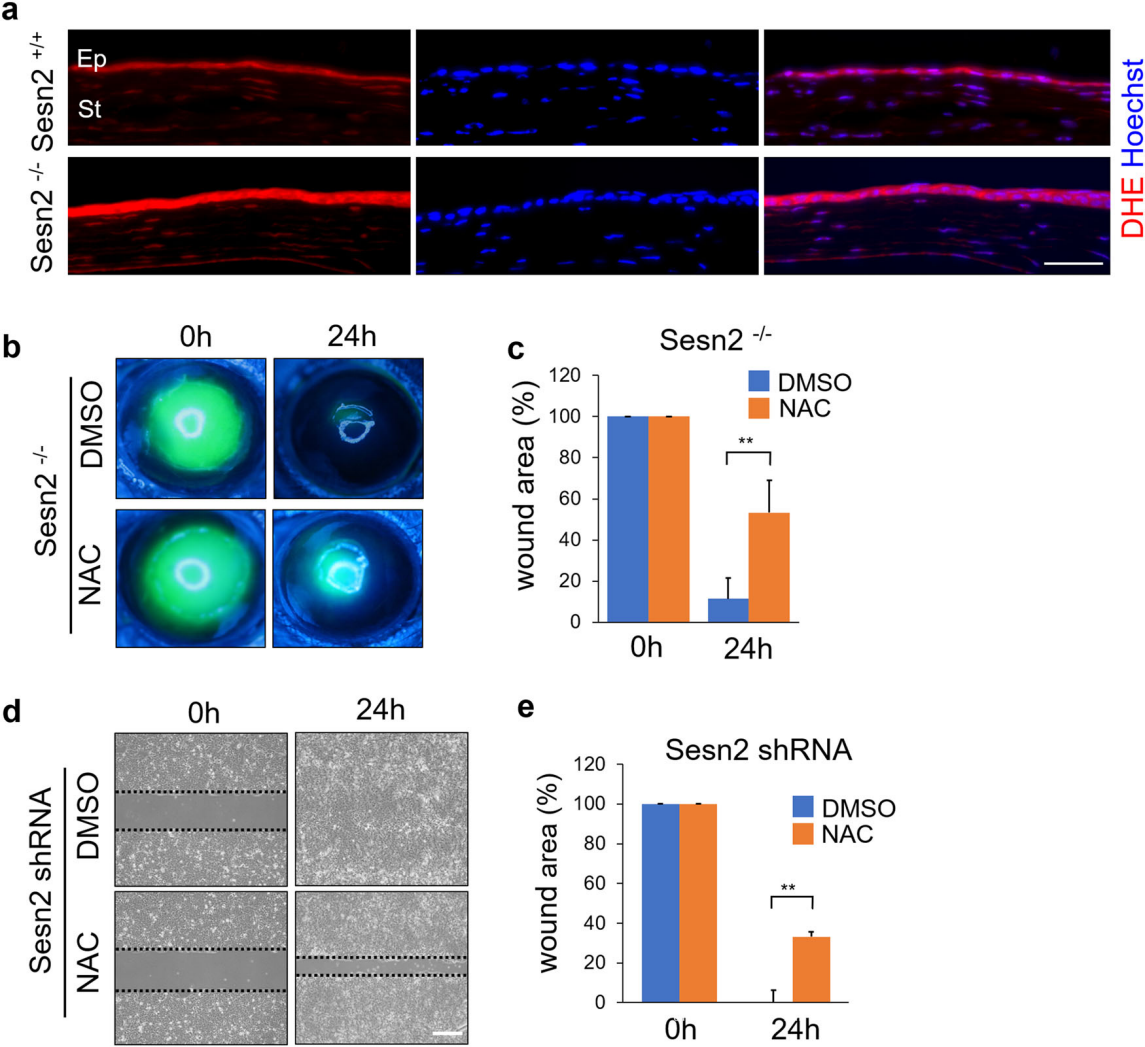
ROS levels increase in Sesn2-deficient corneal epithelium, and the inhibition of ROS suppresses corneal wound healing. **a** Detection of ROS by DHE staining in the sections of the injured corneas from *Sesn2*^−/−^ and *Sesn2*^+/+^ mice. DHE fluorescence intensity in the corneas of *Sesn2*^−/−^ mice was significantly higher than that in the corneas of *Sesn2*^+/+^ mice. **b** In vivo corneal epithelial wound healing of *Sesn2*^−/−^ mice after injury using a 2 mm punch. Each cornea was treated with 2 mM NAC or DMSO after injury. **c** Quantitative analysis of the corneal wound area of *Sesn2*^−/−^ mice treated with NAC or DMSO. Inhibition of ROS suppressed corneal wound healing. **d** In vitro wound healing assay of hCET cells expressing *Sesn2* shRNA treated with 2 mM NAC or DMSO. **e** Wound closure in NAC-treated hCET cells was delayed than that in DMSO-treated cells. Error bars represent the means ± SD of three independent experiments. Two-tailed Student’s *t*-test (**p < 0.01). Scale bar, 50 µm in (**a**); 300 µm in (**d**).

**Fig. 6:**
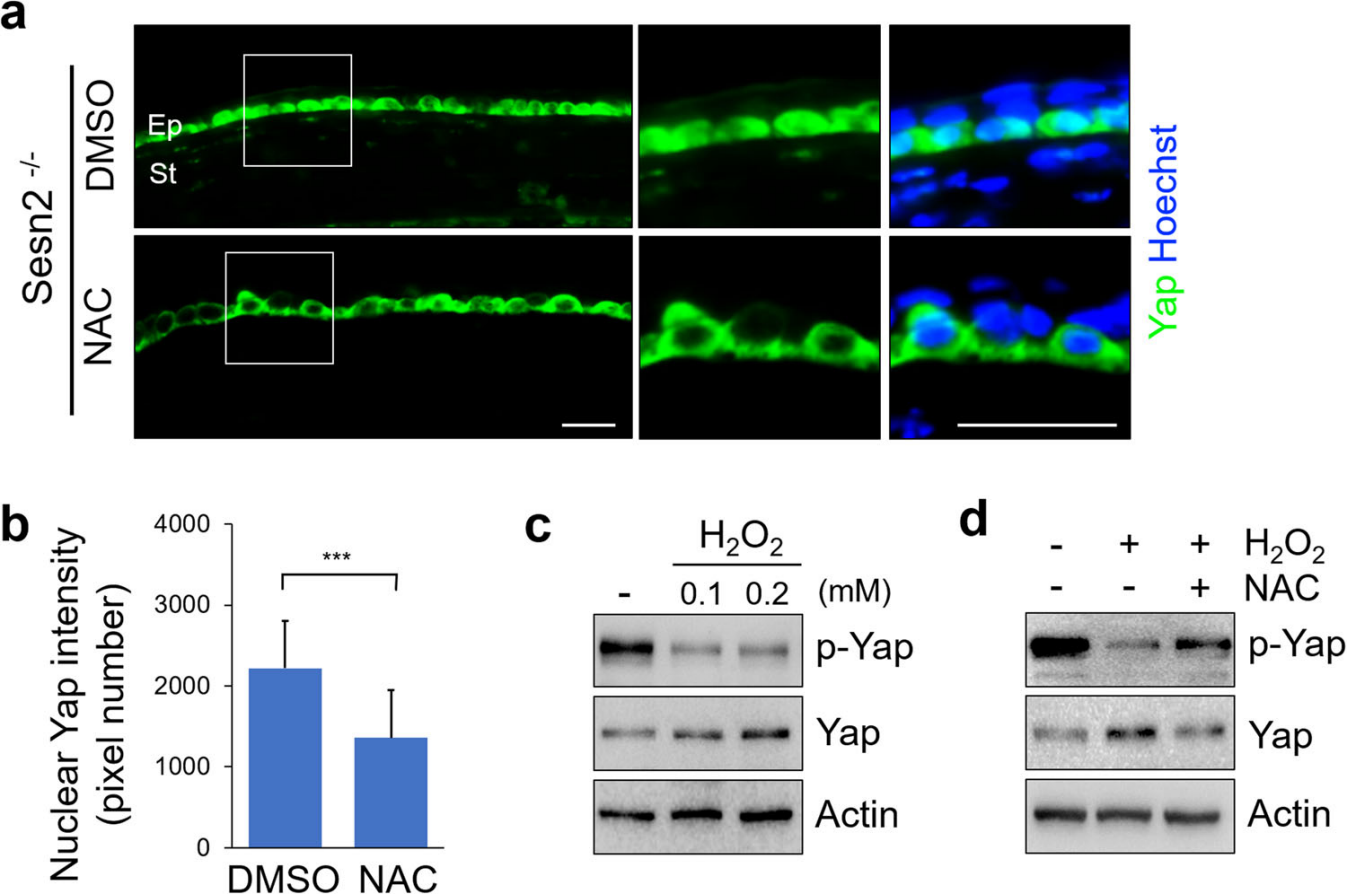
YAP is activated by increased ROS production. **a** Sections of injured corneas from *Sesn2*^−/−^ mice treated with 2 mM NAC or DMSO were immunostained using an anti-YAP antibody. Treatment with NAC decreased YAP nuclear localization. **b** Fluorescence intensity of nuclear YAP in corneal epithelial cells treated with NAC or DMSO. The nuclear YAP fluorescence intensity of NAC-treated corneal epithelial cells was significantly lower than that of DMSO-treated cells. **c** Treatment with H_2_O_2_ increased YAP activity. hCET cells were treated with 0.1 or 0.2 mM H_2_O_2_ for 2 h. Cell lysates were subjected to western blotting with phospho-YAP, YAP, and actin antibodies. **d** Treatment with NAC suppressed YAP activity. hCET cells were treated with 0.2 mM H_2_O_2_ or 2 mM NAC and subjected to western blotting with phospho-YAP, YAP, and actin antibodies. Error bars represent the means ± SD of three independent experiments. Two-tailed Student’s *t*-test (***p < 0.001). Scale bar, 50 µm.

It has been previously reported that Sesn2 suppresses ROS production. To determine whether ROS production is increased in Sesn2-deficient corneas, we stained cells with DHE to specifically detect ROS levels in the injured corneas of *Sesn2*^+/+^ and *Sesn2*^−/−^ mice. The fluorescence intensity for DHE in the corneal epithelial cells of *Sesn2*^−/−^ mice was higher than that of the corneal epithelial cells in *Sesn2*^+/+^ mice, indicating increased levels of ROS in Sesn2-deficient corneas (Fig. 5a). We next treated the injured corneas of *Sesn2*^−/−^ mice with N-acetylcysteine (NAC), an inhibitor of ROS, to evaluate whether ROS promote corneal wound healing. Wound closure of the NAC-treated corneas was delayed as compared to that of DMSO-treated corneas after 24 h (Fig. 5b, c). Moreover, treatment with NAC inhibited wound closure in hCET cells expressing *Sesn2* shRNA, whereas DMSO-treated cells were completely healed after 24 h (Fig. 5d, e). These data suggest that ROS promote corneal wound healing. To investigate whether ROS regulate YAP activity, the injured corneal epithelial cells of *Sesn2*^−/−^ mice were treated with NAC or DMSO and analyzed by immunostaining with an anti-YAP antibody. YAP protein was detected predominantly in the nucleus of DMSO-treated corneal epithelial cells, whereas it was expressed in the cytoplasm of NAC-treated cells (Fig. 6a). Fluorescence intensity of the nuclear YAP in NAC-treated corneal epithelial cells was significantly lower than that in DMSO-treated cells (Fig. 6b). These results indicate that ROS production induces YAP nuclear accumulation. To determine whether ROS regulate YAP activity, we treated hCET cells with H_2_O_2_ and performed western blotting using an anti-phospho-YAP antibody. H_2_O_2_ treatment decreased YAP phosphorylation compared to control (Fig. 6c). The expression level of phospho-YAP protein in hCET cells treated with both H_2_O_2_ and NAC was higher than in hCET cells treated with only H_2_O_2_ (Fig. 6d). Taken together, these results suggest that Sesn2 deficiency increases ROS production and activates YAP in corneal epithelial cells.

## Discussion

Corneal wound healing is essential for the maintenance of corneal transparency and normal visual acuity. Corneal epithelial cell proliferation is a key event in corneal wound healing. However, the molecular mechanisms underlying corneal epithelial cell proliferation are unclear. Here, we identified Sesn2 as a negative regulator of the proliferation of basal epithelial cells in the corneal wound healing process. We also revealed a novel unreported mechanism whereby Sesn2 suppresses YAP activity in an AMPK-independent manner.

Previous studies have demonstrated that Sesn2 suppresses the proliferation of human carcinoma cells, fibroblasts, and epithelial cells^6,29,30^. Herein, we found that Sesn2 is downregulated in epithelial cells during corneal wound healing and that corneal wound healing is faster in *Sesn2*^−/−^ mice than in *Sesn2*^+/+^ mice. Moreover, Sesn2 deficiency in corneal epithelial cells promotes their proliferation. Sesn2 suppresses the mTORC1 pathway, which promotes cell growth and proliferation^31–33^. Sesn2 directly interacts with AMPK, which phosphorylates tuberous sclerosis complex 2 (TSC2) and Raptor and inhibits the mTORC1 signaling pathway^6,34,35^. We showed that phosphorylated S6 was upregulated in Sesn2-deficient corneal epithelial cells in vitro and in vivo. Re-expression of Sesn2 decreased the phosphorylation of S6 protein (Supplementary Fig. S3a). We also found that the inhibition of mTORC1 activity by rapamycin delayed wound closure in the corneal epithelium of *Sesn*^+/+^ and *Sesn2*^−/−^ mice. These results suggest that Sesn2 acts as a negative regulator of proliferation by inhibiting the mTORC1 signaling pathway in corneal wound healing.

YAP plays an important role in epithelial cell proliferation^36–39^. Upon dephosphorylation at the serine 127 site, YAP is translocated into the nucleus and is subsequently activated to promote the transcription of genes associated with proliferation^23,40^. Our results showed that YAP nuclear localization is increased in basal epithelial cells during corneal wound healing and that the number of these cells increased to a greater extent in *Sesn2*^−/−^ mice. Treatment with verteporfin, an inhibitor of YAP, resulted in delayed corneal epithelial wound healing in *Sesn2*^−/−^ mice. Moreover, *YAP* knockdown inhibited the proliferation of Sesn2-deficient hCET cells. These results suggest that YAP activation is required to regulate cell proliferation by Sesn2. A recent study showed that YAP is inactivated upon phosphorylation by AMPK^41^. This result indicates that Sesn2 can potentially regulate YAP activity through AMPK. However, YAP activity was not affected when Sesn2-deficient hCET cells were treated with the AMPK activator AICAR and mTORC1 inhibitor rapamycin, although mTORC1 activity was decreased in these cells. Sesn2 can suppress ROS production through its antioxidant activity^42^. We observed that ROS levels in the corneal epithelium of *Sesn2*^−/−^ mice were higher than those in *Sesn2*^+/+^ mice during corneal wound healing. As signaling molecules, ROS are involved in multiple intrinsic and extrinsic signaling pathways to stimulate cell proliferation^43,44^. A previous study showed that ROS are essential for EGF-stimulated corneal epithelial cell proliferation and wound healing^45^. Our results suggest that ROS can activate YAP to promote proliferation. The expression of phosphorylated YAP protein was decreased upon treatment with H_2_O_2_ to induce ROS production. However, treatment with NAC rescued the expression of phosphorylated YAP protein. Moreover, the nuclear localization of YAP in NAC-treated corneal epithelial cells was decreased with delayed wound closure. Oxidative stress can modulate YAP activity^46^. H_2_O_2_ stimulation induces the binding of TRAF2 to MST1, leading to MST1 activation^47^. MST1/2 are also activated and promote cell death in response to oxidative stress^48,49^. However, YAP forms a complex with FOXO1 and activates FOXO1-mediated transcription of antioxidant genes and subsequently reduces oxidative stress. This process implicates a functional role for YAP in ROS scavenging^50^. Interestingly, a recent study showed increased expression of YAP protein and cell proliferation in neural progenitor cells treated with H_2_O_2_^51^. Another study also reported that elevated intracellular ROS increase YAP protein expression and decrease YAP phosphorylation but does not affect MST1/2 or LATS1^52^. Therefore, we speculate that ROS mediate YAP activation independent of the canonical Hippo pathway in Sesn2-deficient corneal epithelial cells, although the precise mechanism is not clear. In conclusion, our findings indicate that the downregulation of Sesn2 induces YAP activation through ROS production in corneal wound healing, while activation of mTORC1 signaling is mediated by AMPK.

Interestingly, the nuclear localization of YAP was predominantly restricted to the central region undergoing re-epithelialization during wound healing. In contrast, mTORC1 signaling was activated in both the central and peripheral regions. These results suggest that the downregulation of Sesn2 results in the synergistic activation of YAP and mTORC1 signaling to promote re-epithelialization of the corneal wound. A recent study has shown that the expression of YAP in both the cytoplasm and nuclei of basal epithelial cells in the central region and in the peripheral region with epithelial progenitor cells plays a critical role in the maintenance of corneal epithelial progenitor cells^27^. The proliferative potential of corneal epithelial progenitor cells is essential for complete wound repair^1^. Although the precise mechanism regulating YAP activity in corneal epithelial progenitor cells requires further investigation, our findings reveal a novel mechanism for Sesn2-dependent proliferation of corneal epithelial cells involving the functional crosstalk between YAP and the mTOR signaling pathway and suggest that Sesn2 can serve as a potential therapeutic target for re-epithelialization in wound healing.

## Supplementary information

Supplementary information
