# Supplementary material for: Sestrin2 inhibits YAP activation and negatively regulates corneal epithelial cell proliferation
Source: Exp Mol Med. 2020 Jun 12;52(6):951–62. doi: 10.1038/s12276-020-0446-5 (PMC7338388; doi:10.1038/s12276-020-0446-5)
Supplement: Supplementary file 1 — Supplementary information [file 12276_2020_446_MOESM1_ESM.docx]

**Supplementary Information**

**
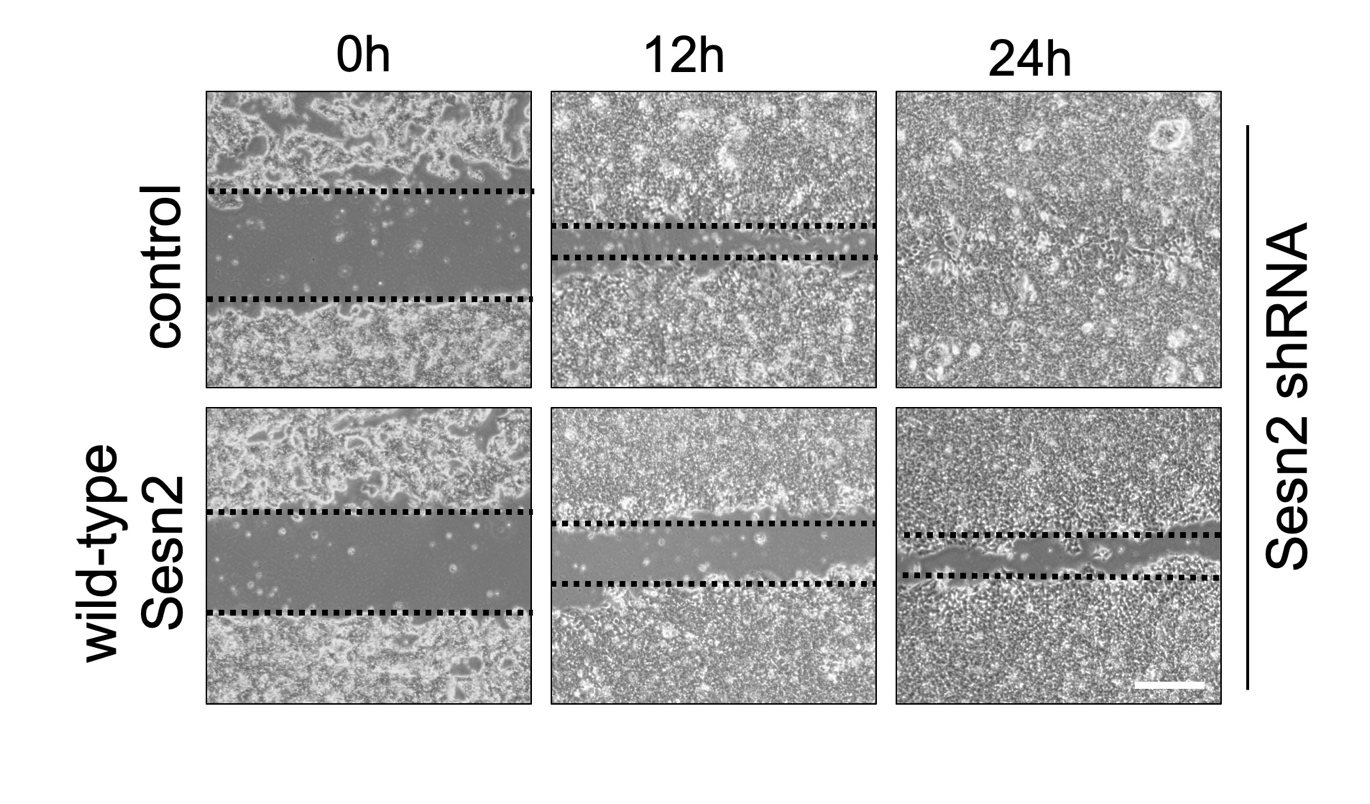
**

**Supplementary Fig. S1. Wound healing seen in Sesn2-deficient hCET cells is suppressed by re-expression of wild-type Sesn2.** *Sesn2* depleted hCET cells were transduced with wild-type Sesn2 and subjected to an *in vitro* wound healing assay. The wound area was photographed immediately at 0, 12, and 24 h. Dotted lines indicate wound borders at given times. Scale bar, 300µm.


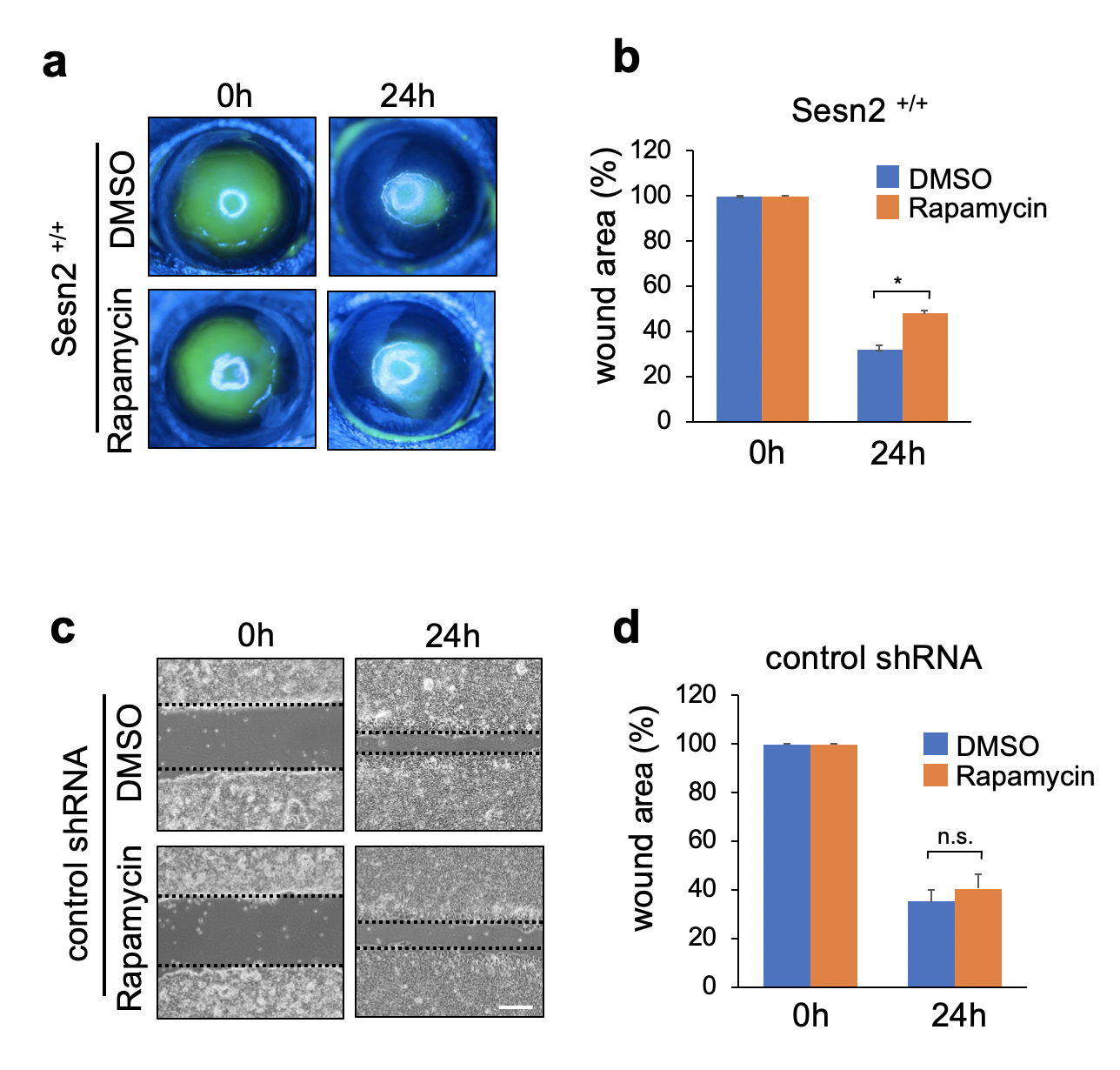


**Supplementary Fig. S2. Effect of rapamycin on corneal wound healing. a** *In vivo* corneal epithelial wound healing in *Sesn2*^+/+^ mice after injury using a 2-mm punch. Each cornea was treated with 100 nM rapamycin and DMSO after injury. At 24 h, the wound area in rapamycin-treated cornea was larger than in DMSO- treated cornea. **b** Quantitative analysis of the wound area in rapamycin- and DMSO-treated corneas shows that rapamycin treatment suppressed the rate of wound closure. **c** *In vitro* wound healing assay of hCET cells expressing control shRNA treated with 100 nM rapamycin and DMSO. **d** Quantitative analysis of the wound area shows no significant difference in the rate of wound closure between control shRNA expressing hCET cells treated with rapamycin and DMSO. Error bars represent the mean ± SD of three or four independent experiments. Two-tailed Student’s *t*-test (n.s.; non-significant, *P < 0.05). Scale bar, 300µm.


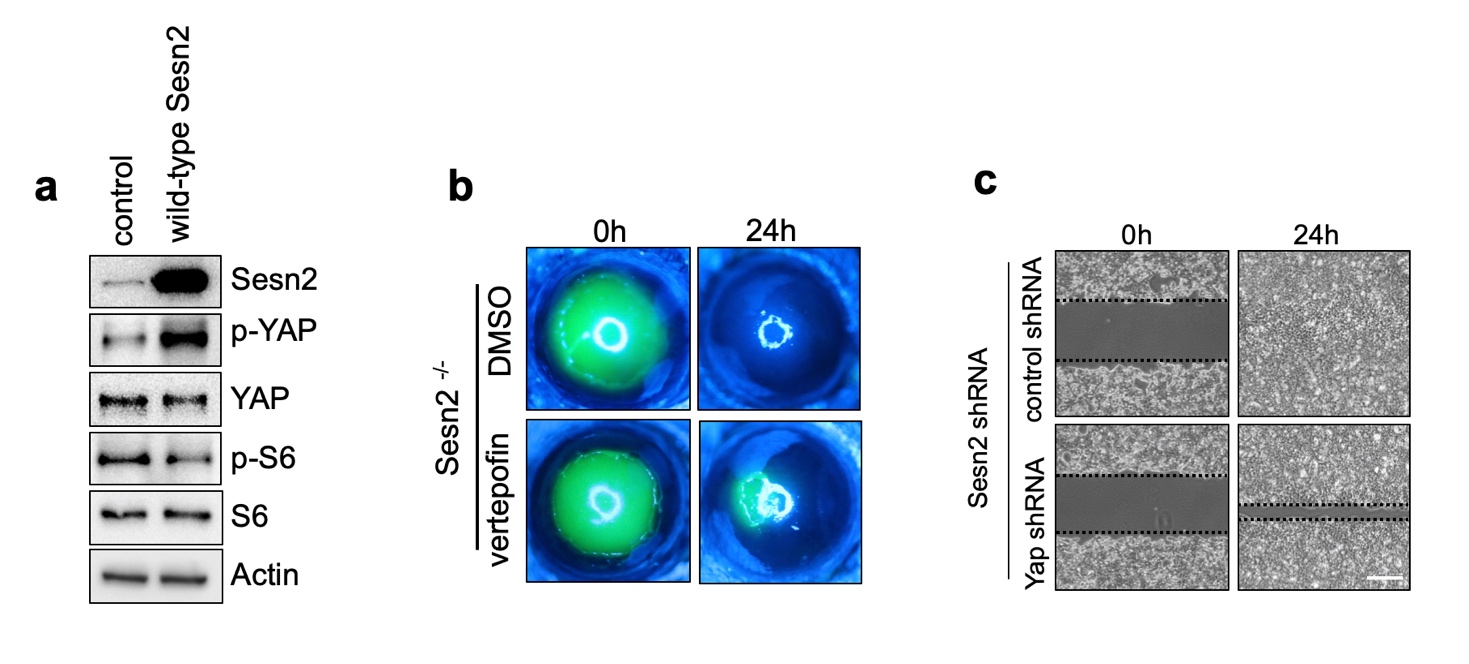


**Supplementary Fig. S3. Sesn2 negatively regulates YAP activity required for corneal epithelial wound healing. a** Ectopic re-expression of wild-type Sesn2 in Sesn2-deficient hCET cells decreases Yap activity and mTOR signaling activity. *Sesn2* depleted hCET cells were transduced with lentivirus expressing wild-type Sesn2. The cell lysates were subjected to western blot with antibodies against Sesn2, phospho-YAP, YAP, phospho-S6, and S6. Western blot analysis shows that re-expression of wild-type Sesn2 increased the expression levels of phosphorylated YAP protein, whereas phosphorylation of S6 was decreased. **b** *In vivo* corneal epithelial wound healing in *Sesn2*^-/-^ mice treated with verteporfin or DMSO. The corneal epithelium was denuded using a 2 mm punch and then treated with 20 µM verteporfin or DMSO. At 24 h after injury, the wound area of verteporfin-treated Sesn2^-/-^ mice remained open, whereas that of DMSO-treated *Sesn2*^-/-^ mice healed. **c** *In vitro* wound healing assay of Sesn2-deficient hCET cells expressing *YAP* shRNA or control shRNA. Scale bar, 300 µm.
